# Advances in PET imaging of ischemic stroke

**DOI:** 10.3389/fstro.2022.1093386

**Published:** 2023-01-18

**Authors:** Paulette D. Orhii, Muhammad E. Haque, Masahiro Fujita, Sudhakar Selvaraj

**Affiliations:** ^1^Department of Psychiatry and Behavioral Sciences, McGovern Medical School, The University of Texas Health Science Center at Houston (UTHealth), Houston, TX, United States; ^2^Department of Neurology, Institute for Stroke and Cerebrovascular Diseases, The University of Texas Health Science Center at Houston (UTHealth), Houston, TX, United States; ^3^Department of Radiology, Houston Methodist Research Institute, Weill Cornell Medical College, Houston, TX, United States

**Keywords:** Positron Electron Tomography (PET), penumbra markers, neuroinflammation, synaptic vesicular protein (SV2A), radioligands, biomarkers, radiotracers

## Abstract

Ischemic strokes make up 87% of all cerebrovascular events. Intravenous tissue plasminogen activator (tPA), a thrombolytic agent, has been recognized as the only viable option for patients with ischemic stroke if administered within 3.5 h of onset and increases the risk of hemorrhagic transformation if administered beyond the treatment window. Acute treatment strategies are centered around rescuing salvageable penumbra. Molecular imaging using positron emission tomography (PET) has shown higher sensitivity and specificity than CT and MRI in delineating penumbral tissues. In addition, PET imaging has identified the role of key inflammatory mediators in atherosclerosis, cellular damage, and recovery. Recently, a novel PET imaging study has shown the feasibility of investigating synaptic density in subacute stroke. Lastly, novel PET radiotracers have been developed to further explore biochemical mechanisms implicated in stroke pathophysiology. Further investigation with PET is needed to understand stroke mechanisms and advance pharmacologic treatment.

## 1. Introduction

### 1.1. Stroke background

Stroke is the number one reason for adult disability in the US and the second leading cause of mortality worldwide (Donkor, 2018). About 87% of stroke cases are classified as ischemic, whereas 13% account for hemorrhagic stroke (Saini et al., 2021). An ischemic stroke occurs when a mass blocks blood vessels in the brain and cuts off cerebral blood flow. Ischemic stroke can be categorized as large vessel atherosclerosis, cardioembolic, lacunar, cryptogenic, or other known etiology (Adams, 1993).

### 1.2. Ischemic stroke pathophysiology

Carotid atheromatous plaques are implicated in up to 20% of ischemic strokes (Evans et al., 2017). The pathophysiology of plaque formation is as follows: damage occurs to the intimal lining of the endovascular wall, which causes monocytes to flood the site of the vessel and release metalloproteinases and fibroblast growth factor (FGF). This causes the creation of an atheroma, which is made up of a necrotic core and a fibrous cap. Atheromatous plaques can cause arterial stenosis or break off and occlude smaller vessels within the cerebral vasculature. The brain, one of the most metabolically active organs in the body, requires a cerebral blood flow of 50 ml/100 g/min to sustain function (Doyle et al., 2008). When blood flow is below 10 ml/100 g/min, the tissue becomes ischemic and prone to cell death (Bernhardt et al., 2017). Significant hypoperfusion over a long enough period will cause necrosis, or irreversible cellular damage, in the ischemic core. Injured cells within the ischemic core release neurotoxins that flood the poorly perfused region surrounding the ischemic core, or the penumbra. In the penumbra, the cells are reversibly damaged and exhibit low electric activity and preserved metabolic function (Astrup et al., 1977). If perfusion is not restored, the penumbra is recruited to the ischemic core. Therefore, the aim of hyper-acute stroke interventions is to preserve penumbral tissue during the hyperacute phase.

The acute phase, which lasts from 24 h to 7 days, is characterized by neuroinflammation and the development of scar tissue. The main inflammatory mediators implicated in the acute phase are leukocytes, microglial cells, adhesion molecules, chemokines, and cytokines such as IL-1, IL-6, TNF*a*, and TGFß (Huang et al., 2006). Interventions are in development to target neuroinflammation during the acute phase of stroke.

The subacute phase of stroke is divided into early (7 days to 3 months) and late (3 months to 6 months). The early subacute stage is marked by increased neuroplasticity; thus recovery aim to restore neural connections. Currently, few interventions are available in patients with late subacute and chronic stroke that can heal the primary damaged tissues and restore motor function. Once the damage has occurred, little can be done to promote neurorestoration. However, spontaneous recovery is known to occur even in the later stages of stroke, and further research could reveal associated biological phenotypes (Bernhardt et al., 2017).

Intravenous tPA and thrombectomy are the only FDA approved treatments for cerebral thromboembolism. Tissue plasminogen activator (tPA), a thrombolytic agent, has been recognized as the only viable pharmacological option for patients with ischemic stroke if administered within 3.5 h of onset. During a hyperacute ischemic event, the rapid neuronal loss urges treatment strategies as “time is brain” (Gomez, 1993). Therefore, the amount of penumbra present at a given time is critical in weighing the risks and benefits of administering tPA. Studies showed that patients treated with tPA within 3–4.5 h of ictus exhibited significantly less disability; however, treatment is often delayed because only 38% of people recognize stroke symptoms (CDC, 2022). Administration of tPA beyond the treatment time window significantly increases the risk of hemorrhagic transformation (Hacke et al., 2008). Imaging is crucial in identifying tPA candidates, staging stroke severity, and monitoring recovery. Recent advancements in molecular imaging have allowed further study into stroke pathophysiology and interventions.

### 1.3. Molecular imaging (MI)

Computed tomography (CT) and magnetic resonance imaging (MRI) are primary diagnostic and differentiating tools between ischemic and hemorrhagic strokes. MRI is non-invasive, cost-effective, and has no risk of radiation and good spatial resolution (Lu et al., 2021). CT perfusion imaging and a combination of MRI diffusion and perfusion-weighted imaging has been extensively applied to identify penumbra.

While MRI and CT are useful in clinical settings, molecular imaging has allowed further understanding of stroke mechanisms, progression, and recovery. Molecular imaging can be very helpful in characterizing various stages of stroke and aiding in better clinical care and rehabilitation. This approach deepens our understanding of various mediators involved in the pathological process, the pharmacodynamics of novel drugs, and key areas for intervention. Using MI, Inoue and Toyoda (2021) showed the potential of extending the endovascular thrombectomy (EVT) from 6 (current recommendation) to 24 hours of onset (Inoue and Toyoda, 2021).

Positron Electron Tomography (PET) is the gold standard for molecular imaging. In PET imaging, radiolabeled ligands for a region of interest (ROI) are intravenously injected into the subject, and uptake is measured with standardized uptake values (SUV) adjusted to the patient's weight and injected dose. At the target site, positrons encounter electrons, resulting in an annihilation reaction and the release of gamma photons. In stroke imaging, PET radioligands have been used to trace and highlight pathologic mechanisms, measure cellular uptake of glucose and oxygen in ischemia, and target cell surface receptors (Evans et al., 2017). PET animal and experimental studies were conducted with ^15^O PET to establish threshold values of ischemic changes (Ackerman et al., 1981; Lenzi et al., 1982; Baron et al., 1984), and new radiotracers for penumbral tissue are in development. In addition to identifying threshold values to characterize stroke lesions, PET imaging has helped to detect plaque vulnerability, identify neuroinflammatory markers in stroke survivors, visualize the pathology of stroke in the subacute and chronic stages, and monitor the effects of therapeutic interventions.

## 2. Major PET-based mechanisms

### 2.1. Imaging of plaque vulnerability

Radioligands have been used to detect high-risk atheromas, which has important implications in the secondary prevention of stroke. The risk of rupture of carotid plaques has been shown to be associated with the plaque's composition, rather than its size or the degree of stenosis of the vessel (Chen and Dilsizian, 2013). MRI has limited sensitivity, so the use of PET imaging in the characterization of plaque morphology has been a crucial advancement in stroke research. FDG was the first radioligand to detect carotid plaques and its uptake was highly correlated with CD68+ macrophage burden (Tawakol et al., 2006). FDG has been shown to be effective in detecting high-risk plaques regardless of the degree of stenosis (Evans et al., 2017). [^68^Ga]-DOTATATE, a radioligand specific for somatostatin receptor subtype 2 highly expressed on macrophages, has also shown promise in detecting symptomatic plaques and has a high correlation to Framingham risk scores (Tarkin et al., 2017). In addition to measuring macrophage burden, PET imaging studies can measure the degree of microcalcification of the fibrous cap, which is implicated in the risk of rupture (Bluestein et al., 2008). [^18^F]-NaF uptake has been shown to be associated with microcalcification, and may be particularly sensitive to identifying an association between aortic atherosclerosis and strokes (Fletcher et al., 2021). Concomitant imaging with [^18^F]-NaF and FDG PET can be used to further assess the risk of carotid atheromas (Chen and Dilsizian, 2013).

### 2.2. Imaging of penumbra

In stroke imaging, PET radioligands have been used to trace and highlight pathologic mechanisms, measure cellular uptake of glucose and oxygen in ischemia, and target cell surface receptors (Evans et al., 2017). PET animal and experimental studies have been conducted with ^15^O PET to identify threshold values of ischemic changes (Ackerman et al., 1981; Lenzi et al., 1982; Baron et al., 1984). CBF, cerebral blood volume (CBV), oxygen extraction fraction (OEF), and cerebral metabolic rate of oxygen consumption (CMRO_2_) are the parameters measured with PET, and can be used to characterize the fate of cells during ischemia. Oligemic cells, which maintain functionality and recover completely, exhibit increased OEF and mildly decreased CBF with preserved CMRO_2_. In the penumbra, CBF is between 12 and 22 ml/100 g/min while OEF is markedly increased and CMRO_2_ is decreased. Finally, in the ischemic core, CBF is below 12 ml/ 100 g/min, CMRO_2_ is below 65 μmol/ 100 g/min and OEF is decreased. The salvageable tissue in the penumbra is therefore identifiable by CMRO_2_. MRI, which has greater accessibility and cost-effectiveness, has largely replaced PET penumbral imaging. However, flumazenil studies have helped to visualize molecular changes characteristic of penumbral integrity that are undetectable by MRI.

[^11^C]-Flumazenil is a marker of selective neuronal loss that has demonstrated utility in mapping ischemic stroke lesions. [^11^C]-Flumazenil binds to cortical GABA-A receptors, which are particularly vulnerable to ischemia (Heiss, 2014). A MRI/PET study showed that areas with reduced CBF and increased [^11^C]-flumazenil uptake corresponded to the area of reversible injury (Heiss, 2014). In addition to identifying penumbra, flumazenil uptake has also been shown to correspond to penumbral susceptibility to secondary damage. This will be discussed further in Section 2.4.

### 2.3. Imaging of neuroinflammation

18 kDa translocator protein (TSPO), a component of glial cells, is an important biomarker of neuroinflammation (Song, 2019). The first TSPO radioligand was [^11^C]-PK11195 was found to have limited specificity (Fujita et al., 2017). Second-generation TSPO radioligands such as [^11^C]-PBR28, [^18^F]-DPA714, and [^18^F]-FEPPA bind with greater specificity, but their uptake is affected by the rs6971 genotype (Fujita et al., 2017). Third-generation TSPO radioligands such as [^11^C]-ER176 have been developed with relatively high specificity (Fan et al., 2016). However, TSPO is also present in endothelial tissue and astrocytes, so it is not specific to microglial inflammation (De Picker and Haarman, 2021). Nonetheless, PET imaging studies have been conducted to evaluate the role of TSPO in stroke pathophysiology. A [^11^C]-PK11195 PET found increased uptake after 72 h within the ischemic core, and after 7 days in the peri-infarct region, ipsilateral hemisphere, and contralateral thalamus (Price et al., 2006). In addition, uptake persisted up to 30 days after the ischemic event. Furthermore, a PET/MRI study found that up to 57% of brain tissue in patients 1–3 years post MCA exhibited uptake in [^11^C]-PBR28 and increased mean diffusion (MD), which suggests that neuroinflammation may be associated with neurodegenerative changes (Schaechter et al., 2021). Additional PET studies conducted on chronic stroke patients found increased uptake in regions distant from the infarcted area (Pappata et al., 2000). The presence of remote inflammation in stroke patients suggests that Wallerian degeneration may be an underlying mechanism in stroke pathology, and there is mixed data regarding whether microglial cells are neuroprotective or neurotoxic in the chronic phase of stroke (Thiel and Heiss, 2011). Microglial inflammation could be a target for intervention in preventing stroke recurrence.

Novel neuroinflammation radiotracers have shown promise in elucidating the role of neuroinflammation in stroke pathophysiology. COX-2 is a product of the arachidonic acid pathway, which plays a key role in inflammation and is the target of non-steroidal anti-inflammatory drugs. COX-2 is particularly important in neuroinflammation, and COX-2 inhibitors have been approved by the FDA for pain management (Minghetti, 2004). Radiolabeled COX-2 inhibitors have been developed to measure COX-2 function, but most demonstrate poor blood-brain-barrier penetration and low affinity to COX-2 receptors (Prabhakaran et al., 2021). However, [^18^F]-MTP, [^11^C]-MCI, and [^11^C]-TMI have shown some promise (Prabhakaran et al., 2021). In addition, radioligands for other neuroinflammation markers such as glycogen synthase kinase 3, monoamine oxidase-B, P2X7 and P2Y12 purinergic receptors, and triggering receptor expressed on myeloid cells-1 (TREM1) are in development (Narayanaswami et al., 2021).

Other neuroinflammation modulators have been shown to have a protective effect in ischemia. One such neuromodulator is the adenosine A1 receptor, which has been shown to regulate the inflammatory response (Joya et al., 2021). A study conducted with [^18^F]-CPFPX demonstrated high specificity for adenosine A1 receptors (Bauer et al., 2003). In a recent animal study, agonism of the adenosine A1 receptor resulted in decreased microglia activation and proliferation post-stroke (Joya et al., 2021), which suggests that the adenosine A1 receptor could play a role in curbing neuroinflammation and preventing infarction.

### 2.4. Imaging of cell injury and death

PET radiotracers are in development to further study the mechanisms of cellular damage. Radiotracers for mitochondrial complex I such as [^1a^F]-BMS, [^1a^F]-BCPP-EF, [^1a^F]-BCPP-BF, [^11^C]-BCPP-EM, and ZCM-I-1 have been developed to study the role of mitochondrial death in the ischemic core and caspase activation in the penumbra during reperfusion (Carinci et al., 2021; Xu et al., 2021). Furthermore, an FDG-PET animal study found remote ischemic preconditioning (RIPC) was protective against mitochondrial caspase activation in ischemic stroke (Lv et al., 2020). A PET animal study conducted with the radiotracer [^11^C]-SAR127303, which is sensitive to the glutamate neuromodulator monoacylglycerol lipase (MAGL), found that administration of minocycline and KML29 could protect the sensorimotor cortex and striatum, respectively (Yamasaki et al., 2021). Such therapies could be used as adjuncts in acute treatment to curb cellular injury. In addition, voltage-gated ions have been studied with FDG-PET and [^1a^F]-3F4AP to explore the roles of gene regulation and demyelination in stroke pathophysiology (Tai et al., 2019; Guehl et al., 2021). Such studies could aid in identifying genetic risk factors in the chronic phase of stroke.

Flumazenil studies have helped image selective neuronal loss in later stroke stages. One study found that survivors of internal carotid or middle cerebral artery strokes had decreased [^11^C]-flumazenil uptake in the thalamus, which was found to be associated with decreased verbal fluency 3 months post-stroke (Yamauchi et al., 2022). Therefore, selective neuronal loss may play a role in chronic stroke symptoms. In addition, selective neuronal loss may occur independently of neuroinflammation in salvaged penumbral tissue: A PET study conducted with [^11^C]-flumazenil and [^11^C]-PK11195, a radioligand for a marker of inflammation further discussed in the next section, found decreased [^11^C]-flumazenil uptake in non-infarcted penumbra with no increase in [^11^C]-PK11195 uptake in acute stroke patients (Morris et al., 2018). Future flumazenil studies could monitor the effectiveness of interventions that target other pathologic mechanisms, such as capillary obstruction and reperfusion injury.

### 2.5. Imaging of synapses

PET imaging has made it possible to study synaptic density or function *in vivo* in humans. Synaptic vesicular protein (SV2A) is expressed in synaptic vesicles throughout the brain and regulates endocytosis and other synaptic vesicle proteins (Yao et al., 2010). SV2A is co-localized with synaptophysin (Finnema et al., 2016), a widely used marker of synaptic density. [^11^C]-UCB-J is a novel PET radioligand that binds to SV2A with high specificity, and has excellent brain uptake and high test-retest reliability. In addition, increased expression of synaptophysin has been found to be associated with motor recovery (Cai et al., 2019).

**Figure 1:**
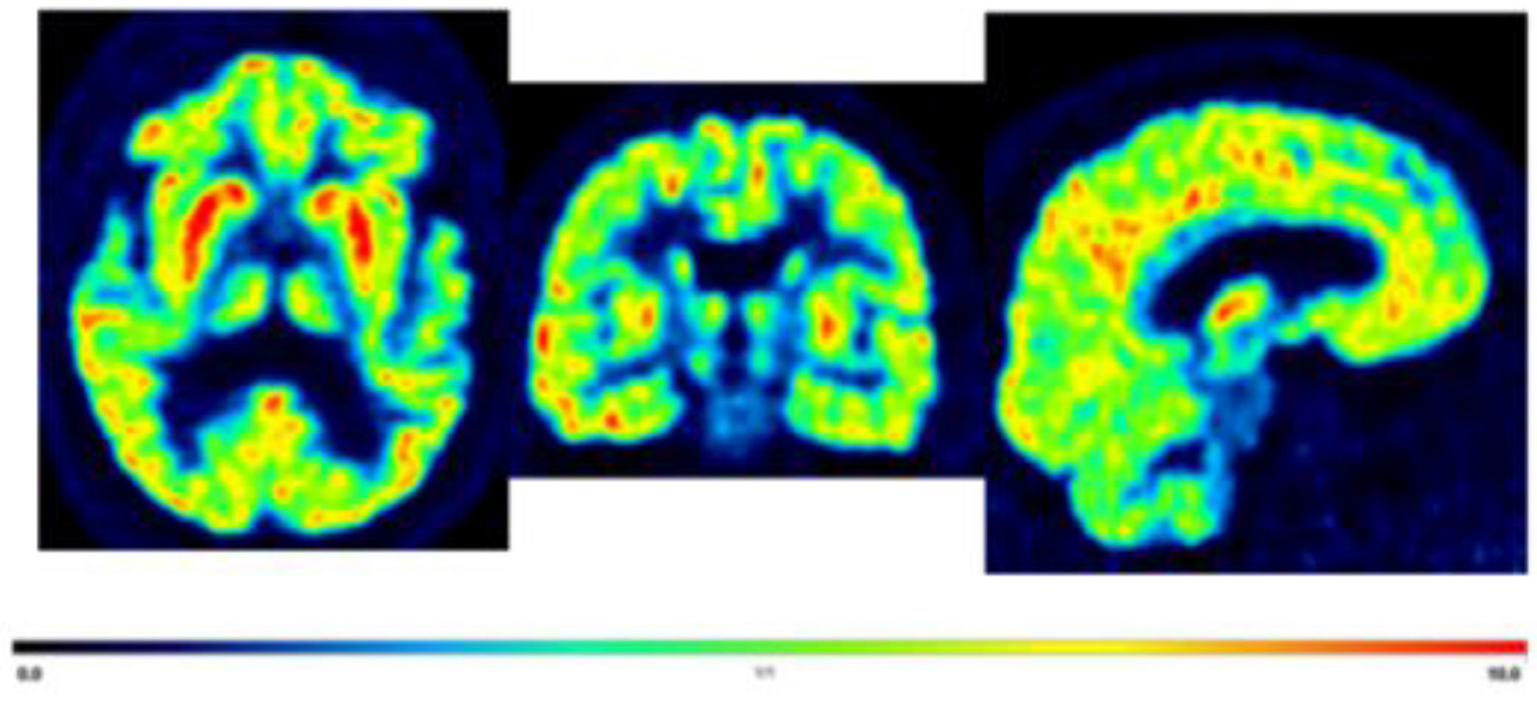
Displaying axial, coronal, and sagittal section of a representative [11C]-UCB-J PET brain image from a 70 years old male with post-stroke depression for illustrative purposes only. Average standardized uptake values (SUV) images from 60 to 90 min. The highest to moderate signal in the cortical, caudate, putamen, and thalamus (red hot color intensity scale).

Studies are being conducted to determine the role of synaptic density in stroke morbidity. A recent PET study using [^11^C]-UCB-J at 3–4 weeks after stroke onset found a decrease of [^11^C]-UCB-J within the lesion of stroke patients and in the non-lesioned tissue of the affected hemisphere compared to the unaffected hemisphere (Michiels et al., 2022). Furthermore, half of the stroke survivors also demonstrated decreased uptake in the contralateral cerebellum, which suggests crossed cerebellar diaschisis (Michiels et al., 2022). In addition, decreased synaptic density has been linked to mood disorders (Leung et al., 2022). Therefore, synaptic loss may be associated with post-stroke depression (Figure 1). While further research is needed, imaging with [^11^C]-UCB-J could help characterize stroke severity and monitor recovery.

In addition, novel radiotracers for synaptic density are being developed. The glutamatergic N-methyl-D-aspartate (NMDA) receptor complex plays a role in neuroplasticity (Paoletti et al., 2013). Studies have shown that genetic mutations in GluN2B, a subunit of the NMDA receptor complex, are protective against ischemic damage (Tang et al., 2018). Animal studies performed with radiotracers such as (R)-[^11^C]-NR2B-Me; (R)-[^18^F]-OF-Me-NB1; (S)-[^18^F]-OF-NB1 have demonstrated acceptable specificity for the GluN2B subunit (Paoletti et al., 2013). Such radiotracers could enable further study into the mechanisms underlying synaptic dysfunction.

## 3. Conclusion

Stroke has a narrow therapeutic window and debilitating long-term effects. Molecular imaging with PET has enabled further study into the *in vivo* biochemical processes underlying ischemic damage at each stage of stroke. Radiotracers such as [68Ga]-DOTATATE and flumazenil have improved the identification of plaque vulnerability and penumbra, respectively. PET studies with novel neuroinflammation radiotracers may expand the breadth of pharmacologic interventions. In addition, PET studies have uncovered the role of selective neuronal loss, a potential target in treating long-term stroke damage. Further, the SV2A radiotracer UCB-J has allowed further study into post-stroke neuroplasticity. These findings have advanced stroke treatment by facilitating clinical staging, prognosis, disease monitoring, and treatment. Further study with PET can identify new biological targets, vulnerable phenotypes, and novel therapeutics.

## Author contributions

SS developed the concept and design of the manuscript. PO completed the first draft. MH and MF contributed to the editing of the manuscript. All authors read and approved the final manuscript.
